# Towards an Optimized Distributed Message Queue System for AIoT Edge Computing: A Reinforcement Learning Approach

**DOI:** 10.3390/s23125447

**Published:** 2023-06-08

**Authors:** Zaipeng Xie, Cheng Ji, Lifeng Xu, Mingyao Xia, Hongli Cao

**Affiliations:** 1 Key Laboratory of Water Big Data Technology of Ministry of Water Resources, Hohai University, Nanjing 211100, China; 2 Department of Computer Science and Technology, Hohai University, Nanjing 211100, China; chengji@hhu.edu.cn (C.J.); lifengxu.hhu@hhu.edu.cn (L.X.); mingyao.xia@hhu.edu.cn (M.X.); 3 Key Laboratory of Underwater Acoustic Signal Processing of Ministry of Education, Southeast University, Nanjing 210096, China; honglicao@seu.edu.cn

**Keywords:** artificial intelligence of things, distributed message queue, reinforcement learning approach, AIoT edge computing, system throughput performance

## Abstract

The convergence of artificial intelligence and the Internet of Things (IoT) has made remarkable strides in the realm of industry. In the context of AIoT edge computing, where IoT devices collect data from diverse sources and send them for real-time processing at edge servers, existing message queue systems face challenges in adapting to changing system conditions, such as fluctuations in the number of devices, message size, and frequency. This necessitates the development of an approach that can effectively decouple message processing and handle workload variations in the AIoT computing environment. This study presents a distributed message system for AIoT edge computing, specifically designed to address the challenges associated with message ordering in such environments. The system incorporates a novel partition selection algorithm (PSA) to ensure message order, balance the load among broker clusters, and enhance the availability of subscribable messages from AIoT edge devices. Furthermore, this study proposes the distributed message system configuration optimization algorithm (DMSCO), based on DDPG, to optimize the performance of the distributed message system. Experimental evaluations demonstrate that, compared to the genetic algorithm and random searching, the DMSCO algorithm can provide a significant improvement in system throughput to meet the specific demands of high-concurrency AIoT edge computing applications.

## 1. Introduction

The advancements in artificial intelligence (AI) have led to the widespread deployment of AI applications, ranging from Industry 4.0 [1] to smart cities [2]. With the growing adoption of the Internet of Things (IoT) and edge computing, IoT devices used in these applications generate vast amounts of data that require real-time processing by servers. The combination of AI and IoT has given rise to Artificial Intelligence of Things (AIoT) systems [3,4,5,6], which can enhance IoT operations, improve human–machine interaction, and optimize data management. However, conventional message queues utilized in IoT systems are optimized for lightweight publish/subscribe messaging, rendering them inadequately equipped to manage the extensive AIoT message transmission effectively. AIoT systems may also face message floods due to network jitter or failures, necessitating an ideal message queue that can handle volatile environmental factors smoothly. Several message queue systems [7,8,9,10,11,12] have been developed for IoT data processing. However, applying these message queues in the dynamic AIoT computing environment poses challenges. The varying network conditions, device numbers, message sizes, and frequencies require methods to optimize the performance of distributed message queues for AIoT systems. It is crucial to overcome limitations imposed by hardware resources and meet the low latency and timing requirements of real-time AIoT systems [11].

Distributed message queues are essential in various data processing application scenarios, providing users with configurable parameters to customize their performance. For instance, both RabbitMQ and Kafka offer over one hundred adjustable parameters [13,14], which often require manual adaptation [15] to suit different message transmission scenarios. However, the sheer number of parameter combinations makes it practically impossible to search for an optimized solution within a limited time for the system’s varying scenarios. As a result, users often resort to applying a default configuration when setting up the parameters [15]. Therefore, an algorithm is desired to effectively optimize the performance of distributed message queues in AIoT edge computing scenarios. This algorithm would be capable of adaptively selecting and fine-tuning critical parameters that may have a significant impact on throughput.

**Figure 1:**
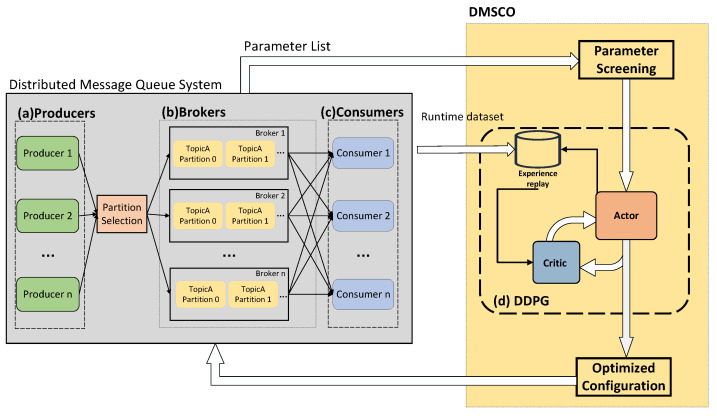
System diagram of the proposed DMSCO algorithm system. (**a**) Producers convert data or streams into messages and send them to brokers. (**b**) Brokers store and manage the messages. (**c**) Consumers receive and process the messages. (**d**) The gathered run-time metric is utilized by DDPG to train the agent’s policy network.

This study proposes a distributed message system for large-scale AIoT based on Kafka to address message ordering challenges in AIoT edge computing. A novel partition selection algorithm (PSA) is introduced to maintain the AIoT message order, balance the load among broker clusters, and augment availability during the publication of subscribable messages by AIoT edge devices. Then, a DDPG-based distributed message system configuration optimization algorithm (DMSCO) is proposed for the proposed AIoT message queue systems. The overall diagram of the proposed DMSCO algorithm is illustrated in Figure 1. In the targeted edge AIoT scenario of the distributed message system, four distinct processes operate on each of the three types of devices. Firstly, producer processes execute on end devices, such as sensors and cameras, with the primary function of converting raw data into messages for transmission. Secondly, the broker process, responsible for processing and storing incoming messages, runs on the edge server. Thirdly, the consumer process operates on the edge host, enabling the consumption of readily available messages. Lastly, the optimization process, also running on the edge server, collects operational metrics of the system and performs the necessary optimizations.

The DMSCO algorithm is a four-phase process that optimizes our AIoT distributed message system. The first phase involves obtaining a parameter list that includes all adjustable variables. In the second phase, principal component analysis (PCA) [16] is utilized to reduce the dimensionality of the parameter list. The third phase involves filtering the preprocessed parameter list using Lasso regression [17] to identify significant variables and build a performance model mapping input configurations to performance metrics. In the fourth phase, the optimization process is initiated by passing the runtime metrics dataset to the reinforcement learning model and dynamically optimizing using the deep deterministic policy gradient (DDPG) algorithm. In summary, our contributions are as follows

- We propose a distributed message system for large-scale AIoT based on Kafka to address message ordering challenges in AIoT edge computing. The impact of different factors on system performance in distributed AIoT messaging scenarios is investigated. A partition selection algorithm (PSA) is specifically designed for the proposed distributed message queues, aiming to maintain the order of AIoT messages, balance the load among broker clusters, and enhance availability during the publication of subscribable messages by AIoT edge devices.
- We propose a reinforcement-learning-based method called DMSCO (DDPG-based distributed message queue systems configuration optimization) that utilizes a preprocessed parameter list as an action space to train our decision model. By incorporating rewards based on the distributed message queue system’s throughput and message transmission success rate, DMSCO efficiently optimizes messaging performance in AIoT scenarios by adaptively fine-tuning parameter configurations.
- We conducted a comprehensive evaluation of the proposed DMSCO algorithm, assessing its performance efficacy for the distributed message queue system in AIoT edge computing scenarios across varying message sizes and transmission frequencies. Through comparative analysis against methods employing genetic algorithms and random searching, we observed that the DMSCO algorithm provides an improved solution to meet the specific demands of larger-scale, high-concurrency AIoT edge computing applications.

The remainder of this paper is structured as follows: Section 2 provides an overview of related work on distributed message queues and their optimization. Section 3 details the proposed partition selection algorithm for the distributed message queue. In Section 4, we describe the reinforcement learning algorithm used to optimize the performance of the message queue. Section 5 discusses the conducted experiments and analyzes the proposed algorithms in terms of message transmission success rate and system throughput. Finally, Section 6 presents the conclusions drawn from this study.

## 2. Related Work

The field of distributed message queues has garnered significant attention from both industry and academia. There are quite a few messaging protocols [18,19] available to choose from for various types of requirements of IoT systems. For example, message queuing telemetry transport (MQTT) [8] is a lightweight messaging protocol for IoT systems, known for low bandwidth and power consumption, reliable message delivery, and a flexible publish/subscribe model. It is suitable for resource-constrained devices but has limitations in handling large amounts of data and lacks built-in security. In addition, its centralized broker architecture can be a single point of failure in large deployments. Constrained application protocol (CoAP) [20] is a lightweight communication protocol for IoT devices and networks with limited resources. It enables efficient data exchange and resource management and uses UDP to transport compact message formats and multicast communication. Advanced message queuing protocol (AMQP) [19] is another standard open protocol that enables both publish/subscribe and point-to-point messaging patterns between applications or systems. Both MQTT and AMQP are robust and widely adopted in various messaging implementations [19].

Additionally, while HTTP protocol can be used in certain IoT scenarios [21] due to its widespread usage and support for request–response interactions, it may be inefficient for resource-constrained devices with limited bandwidth and power. Its client–server model and lack of native support for lightweight publish/subscribe messaging and asynchronous communication make it less optimal for IoT environments that require peer-to-peer or decentralized communication. In such cases, specialized protocols such as MQTT, CoAP, or AMQP are often preferred. Table 1 presents a comparison of popular distributed messaging protocols for IoT networks, including MQTT, CoAP, AMQP, and HTTP.

The development of message queues has led to the creation of various implementations that support different protocols. RabbitMQ [13] is an enterprise-level message queue designed to handle high-concurrency scenarios and offers good performance with low latency, enabling efficient message processing. RabbitMQ supports MQTT, CoAP, AMQP, and HTTP protocols, making it compatible with a wide range of IoT systems. RocketMQ [11], on the other hand, supports publish/subscribe and point-to-point messaging patterns and can be integrated with MQTT, CoAP, and HTTP protocols. However, RocketMQ’s deviation from the Java Message Service specification has led to limited adoption in the industry due to challenges associated with system migration. ActiveMQ [7] supports MQTT, AMQP, and HTTP protocols but has a centralized architecture and limited single-machine throughput performance, which has contributed to its recent decrease in popularity.

Apache Kafka [12] stands out as a distinct messaging system that emphasizes scalability, durability, and efficient data processing. Kafka’s architecture is built around a distributed commit log that enables multiple consumers to read and process data simultaneously, making it suitable for real-time analytics, event streaming, and data integration use cases [11]. In the context of AIoT systems, Kafka’s scalability and fault-tolerant nature make it well-suited for handling the massive data streams generated by AIoT devices, including sensor data, telemetry information, and events from distributed IoT networks. Its support for protocols, including MQTT and HTTP, allows seamless integration with IoT devices, enabling real-time data ingestion, processing, and analysis. However, challenges [22] exist in adapting Kafka to evolving AIoT message transmission scenarios, ensuring message ordering between partitions, and addressing issues related to repeated message consumption. Researchers [9,14,15,23] have been actively working on improving Kafka’s cluster development, operation, reliability, and maintenance costs. Despite its strengths, Kafka relies on ZooKeeper for coordination management, and practical message partition selection algorithms and adaptive performance optimization remain open challenges.

Several optimizations methods [24,25,26,27,28] are discussed to improve the performance of distributed systems. Donta et al. [24] summarize various message queues and message brokers used in IoT systems, and they find out that multiple message queues handle messages as per predefined constraints, making them static in nature. The authors further argue that existing auto-configuration methods, which rely on abstract models derived from software architecture, can be prone to inaccuracies and may not effectively adapt to changing conditions. Dou et al. [25] introduce HDConfigor, an automatic configuration tuning tool designed for log search engines such as Elasticsearch. These search engines typically expose many configuration parameters related to performance, and HDConfigor aims to streamline the process of configuration tuning, reducing both time and labor requirements. HDConfigor utilizes the modified random embedding Bayesian optimization algorithm (mREMBO) to create a low-dimensional embedded space through a random embedding matrix. It then performs Bayesian optimization within this space, resulting in an additional 10.31% improvement in throughput. Ma et al. [26] propose a new message queue architecture, NetMQ, that utilizes programmable switches to handle message production and consumption requests for high-demand queues efficiently. A heuristic algorithm is utilized to update the cached topic partition regularly, which helps to improve the throughput performance for dynamic workloads. However, NetMQ is designed for rack-scale message queue systems and may not be suitable for all types of message queue applications. Additionally, implementing NetMQ may require specialized hardware and expertise in programmable switches. Dou et al. [27] propose DeepCAT for online configuration auto-tuning for big data frameworks. DeepCAT leverages the TD3 (twin delayed deep deterministic policy gradient) algorithm and incorporates a novel reward-driven prioritized experience replay mechanism. It also utilizes a Twin-Q optimizer to estimate execution time. Experimental results conducted on a local Spark cluster with HiBench benchmark applications showcase the effectiveness of DeepCAT in achieving improved performance with reduced tuning costs. Recently, Dou et al. [28] propose a cost-efficient approach called TurBO that enhances Bayesian optimization (BO) to handle sub-optimal configurations for big data-processing frameworks. Their experimental evaluations on a local Spark cluster demonstrate that TurBO outperforms three representative baseline approaches, achieving significant speedup in the tuning process.

However, most existing methods for optimizing distributed systems in AIoT application scenarios have limitations. These methods often rely on collecting configuration status to identify performance bottlenecks, which can be time-consuming, especially in scenarios with a large state space. Additionally, the existing distributed message queues lack the ability to adapt to dynamic network conditions, such as fluctuations in the number of devices, variations in message size, and changes in sending frequency. On the other hand, learning-based methods typically require a sparse prediction model obtained through sampling the high-dimensional configuration space for optimization. It remains unclear whether these methods are suitable for effectively optimizing the real-time processing of distributed message queues in AIoT edge computing, where message scales frequently change. Therefore, it is imperative to research optimization methods that can address these challenges and improve the performance of distributed message queues in AIoT edge computing.

## 3. Distributed Message Queue System for AIoT Edge Computing

The primary focus of performance optimization for conventional distributed message queues lies in the transmission of messages from end devices to centralized servers in cloud computing settings [29]. However, in AIoT edge computing environments, there is a lack of adequate evaluation and optimization for message transmission and processing within edge devices and edge-to-edge communications. These environments involve varying numbers of end devices and message scales. For instance, sensors used for simple monitoring generate small data packages, while scenarios such as robot collaboration, smart cities, drones, and autonomous driving require high message transmission volumes across numerous end devices. Moreover, distributed federated learning with AIoT poses a significant challenge due to generating a considerable volume of messages between end devices, including model updates and parameters. Therefore, the distributed message queues must handle varying computational capabilities and network connectivity among end devices, which may lead to delays or timeouts during message transmission. Thus, the distributed message queue system must be designed with high throughput, scalability, and availability for efficient operation to manage these scenarios.

### 3.1. Distributed Message System for Large-Scale AIoT Edge Computing

Traditional point-to-point message transmission often faces challenges [30] such as network congestion and a high message loss rate in large-scale scenarios. To address these challenges, several message queue solutions have been developed. These distributed solutions are usually designed with architectures tailored to specific scenarios. For instance, Kafka, a widely used distributed message queue system, consists of several key components [15], including producers, consumers, brokers, topics, partitions, and zookeeper. Producers are the data source, publishing it into topics, which are streams of related messages. Consumers then receive this data, subscribing to relevant topics. Topics are further divided into partitions for better organization and performance, with each partition stored on one or more brokers—servers responsible for maintaining and coordinating Kafka’s data. Finally, Zookeeper acts as the management backbone of Kafka, handling service discovery for the brokers and coordinating leader elections for partitions.

In AIoT edge computing, ensuring message ordering is of utmost importance when transmitting messages between various AIoT edges and data centers. If the order of messages gets disrupted, it could lead to incorrect data analysis, ineffective decision-making, and potential system disruptions. For this purpose, we propose implementing a distributed message system for large-scale AIoT based on Kafka, where data reading and writing occur at the partition level. While maintaining message order within a single partition is a strength of Kafka, it should be noted that relying solely on a single partition can limit throughput and reduce load-balancing capabilities. It is generally advisable to leverage Kafka’s multi-partitioning capabilities for improved performance and scalability in large-scale AIoT deployments. However, it is important to note that, when multiple partitions are utilized, Kafka only guarantees the ordered consumption of messages at the partition level, leaving the ordering between partitions uncertain. Hence, it is necessary to address the challenge of message disorder among partitions when employing a multi-partition strategy.

In our proposed partition selection algorithm (PSA) for distributed message queues, our objective is to enhance functionality in scientific applications by addressing the limitations of two default partition strategies. The primary strategy assigns a unique key to each message, ensuring that messages with the same key are stored in the same partition for systematic organization. The secondary strategy employs a round-robin approach, allocating partitions sequentially and caching messages incrementally from the initial to the final partition. Nonetheless, traditional Kafka systems’ default partition strategies pose a trade-off. The first strategy guarantees message order but may result in congestion within specific partitions. Conversely, the second strategy strives to distribute the load evenly across partitions without ensuring a strict message order. Our tailored PSA aims to surmount these challenges and achieve equilibrium in distributed message queues for scientific applications.

Algorithm 1 presents the proposed PSA scheme designed to address the unique characteristics of messages in the AIoT edge computing environment and prevent message disorder resulting from polling strategies. Our PSA follows a specific approach for directing messages based on their key. If a corresponding partition exists for the key, the message is directed to that specific partition. However, if no association exists and there are available partitions (where the number of available partitions is denoted as m>0), a hash function is applied to the message key to determine an accessible partition. The remainder determines the target partition when dividing by m+1. In cases where *m* equals zero, the remainder is computed by dividing by n+1, where *n* represents the total number of partitions. By implementing this customized partition selection approach, our proposed message system for large-scale AIoT can overcome the limitations of default strategies, ensuring efficient message distribution, load balancing, and maintaining the desired message order whenever feasible.
**Algorithm 1:** Partition selection algorithm (PSA).
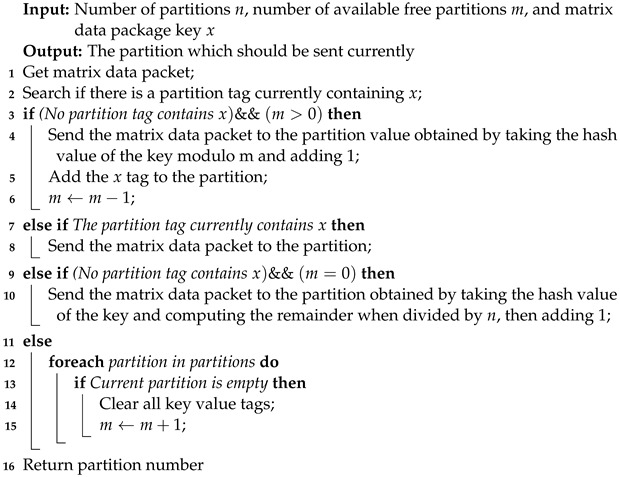


    The proposed PSA algorithm plays a crucial role in our messaging system. It effectively distributes a large volume of messages, sent simultaneously to different terminals, among the available partitions. This strategy ensures a balanced workload across the system, achieving optimal load balancing. Additionally, PSA maintains the order of messages destined for the same AIoT destination, mitigating the detrimental effects of message disorder within the distributed message system. This approach guarantees reliable and efficient message processing.

### 3.2. Performance Modeling in AIoT Edge Computing Scenarios

We assess the performance of distributed messaging systems in large-scale settings by simulating a distributed environment comprising multiple edge devices. Each device in the system serves a dual role, functioning as both a producer and a consumer. The procedure within each edge device encompasses four distinct processes. In the first process, matrices of varying sizes are generated according to a normal distribution, provided that the local queue is not at capacity, effectively simulating real-time message generation. In the second process, messages from other devices are consumed, resulting in the creation of verification data packets. The third process assesses the successful delivery of validation messages by examining the hash table and the received verification data packet. Finally, a dedicated process analyzes network packets to ascertain their association with matrices or verification data packets. This comprehensive approach ensures a smooth and logical evaluation of distributed messaging systems in large-scale environments, providing valuable insights for further research and development.

**Figure 2:**
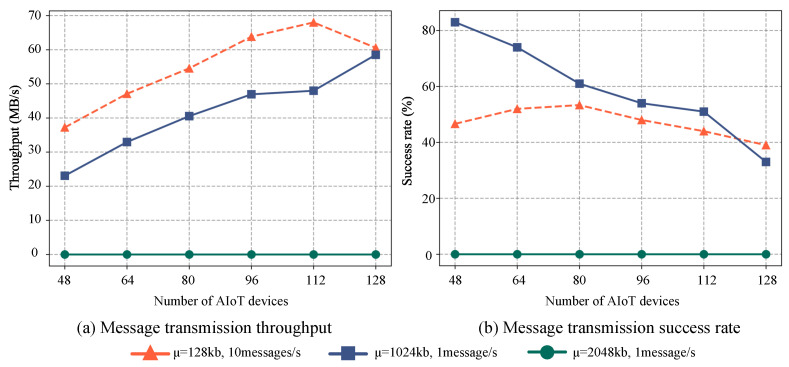
The performance of message transmission with different quantities and characteristics varies as the number of AIoT edge devices increases. (**a**) The throughput variation and (**b**) the success rate variation.

To evaluate the performance of the distributed message systems, we conduct comparisons under various scenarios with differing numbers of devices and running times. The experiment divides devices into two groups, each transmitting messages at different rates and sizes. One group transmits ten messages per second, where each message size follows a normal distribution with a mean of μ=128 KB and a standard deviation of σ=10. Conversely, the second group transmits a single message per second, with each message size conforming to a normal distribution with a mean of μ=1024 KB or 2048 KB, and a standard deviation of σ=200. Message transmission effectiveness is evaluated based on success rate and throughput over a duration of 60 min, with results depicted in Figure 2. These distinct groups reflect two disparate messaging characteristics: high frequency for small messages and low frequency for large messages. The former is suitable for real-time applications such as chat applications, intelligent sensor data monitoring, and gradient passing in distributed machine learning, where rapid, frequent updates are crucial. The primary challenge here is maintaining low latency while handling high volumes of small messages. In contrast, the low-frequency, large-size messages are appropriate for applications requiring extensive data transfer such as file sharing, sensor video streaming, and data backup. Here, the challenge is ensuring efficient and reliable data transfer amidst network congestion and bandwidth constraints.

Experimental results indicate a significant decrease in the success rate of the distributed message system as the number of devices increases, particularly when transmitting frequent small messages. This decline can largely be attributed to insufficient sending and receiving buffers, leading to substantial losses during message transmission between the message queue and the consumer. However, the overall system throughput increases with a larger number of devices, peaking at 68.5 MB/s. This throughput increase is primarily due to the system’s ability to handle a larger volume of messages simultaneously with the integration of more devices. Even though the success rate of individual devices may decline, the system’s capacity to process more messages overall enhances the throughput. As is shown in Figure 2, the proposed PSA enhances load-balancing by efficiently distributing a large volume of messages sent to different edge devices among available partitions. It also ensures the orderly delivery of messages to individual edge devices, effectively mitigating the negative impacts of message disorganization on the distributed messaging system.

For less frequent messages with an average size of 1024 KB, the success rate of message transmission is approximately 50%, despite some devices encountering message errors. This can be explained by the default maximum value for single messages being set at 976 KB. Given the normal distribution of message sizes (mean of 1024 KB and standard deviation of 200), roughly half of the messages exceed the default maximum value. Moreover, when the average size of individual messages is 2048 KB, both the success rate and throughput of the system fall drastically, with all devices displaying message-sending error prompts due to the messages surpassing the maximum value.

These findings suggest that the proposed distributed message queue is suitable for IoT message transmission scenarios among devices in edge environments. However, its performance may not be optimal under default configurations, particularly for high-concurrency message transmission scenarios in AIoT edge computing environments. Traditional distributed message queues may not be able to dynamically adjust and optimize message transmission mechanisms based on the current state, such as the number of devices, message size, and transmission frequency. To address this issue, we propose a distributed message system configuration optimization (DMSCO) approach. This approach leverages reinforcement learning to optimize the parameter configuration of distributed message queues, aiming to improve the efficiency and effectiveness of message transmission in dynamic and resource-constrained AIoT environments.

## 4. Reinforcement-Learning-Based Method for Optimized AIoT Message Queue System

The AIoT environment often requires real-time data processing capability with low latency. However, conventional message queue systems can lead to message loss, network congestion, and insufficient real-time processing capacity. Existing distributed message systems, which rely on message queues for message delivery, lack the flexibility to optimize message delivery mechanisms in accordance with variations in device numbers, message sizes, and frequencies [15].

In order to develop an efficient optimization approach for AIoT systems, we start by investigating the relationship between distributed message queues and their essential parameter configurations. We identify a set of 22 critical parameters, along with their respective data, which significantly influence the performance of producers and brokers in our proposed Kafka-based messaging queue system. Then, we employ the PCA to reduce dimensionality through data preprocessing. After that, we utilize Lasso regression to construct an optimization model using the resulting parameters to be optimized. Lasso regression [17] is employed due to its ability to perform feature selection and regularization. It reduces the impact of less important parameters by shrinking their coefficients towards zero, aiding in dimensionality reduction, overfitting prevention, and model interpretability. It effectively performs feature selection, allowing us to identify the most significant parameters that influence the performance of the distributed messaging system.

**Figure 3:**
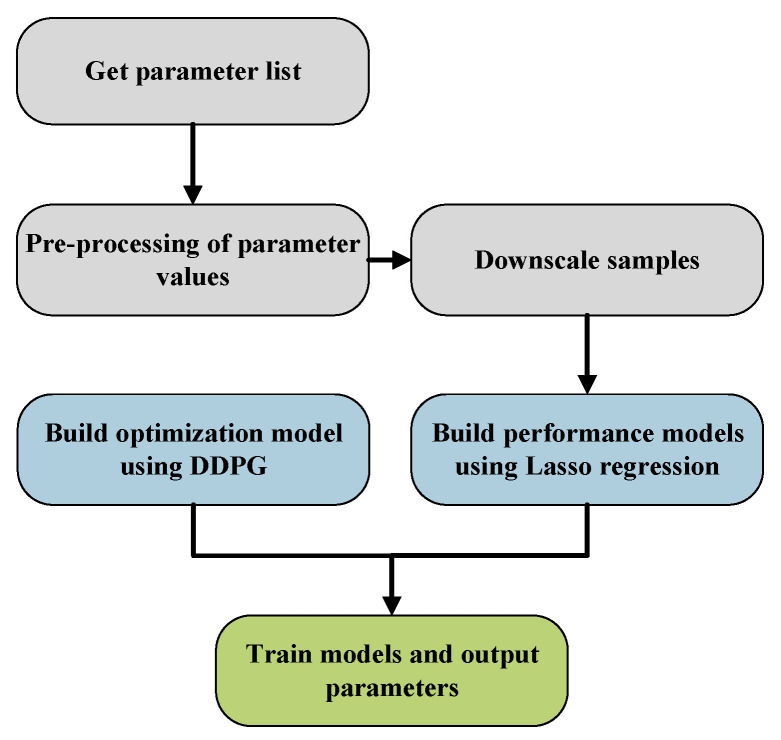
Performance optimization process diagram.

Subsequently, we use the deep deterministic policy gradient (DDPG) method [31] to optimize the parameters of the proposed distributed message queues. This approach allows us to maximize the system throughput under the current message scale, achieving adaptive optimization of system performance. Figure 3 provides an illustration of the optimization process.

### 4.1. Parameter Screening

In this study, the parameter configuration for the distributed message queue can be adjusted using a configuration file. The parameters that potentially influence the throughput performance of the distributed message queue are included within the broker and producer configurations. Upon reviewing the technical manual and parameter descriptions, we have identified 22 parameters among the numerous available options that are most likely to impact the throughput performance significantly.

We classify the parameter types into discrete and continuous to generate samples for the selected parameters that may impact the performance of distributed message queues. Discrete parameters include categorical and discrete numerical variables. For instance, “cType” is a categorical variable with values such as “uncompressed”, “producer”, and “gzip”. Conversely, “bThreads” and “rTMs” are numerical variables, with “bThreads” being a discrete numerical variable and “rTMs” being a continuous numerical variable. For each variable, we select three possible values: K0 (below default), K1 (default), and K2 (above default). These values aim to capture the characteristics of the parameter and ensure a comprehensive evaluation of its performance under different settings.

Once the parameter values are selected, they undergo preprocessing. The parameters of the distributed message queue are categorized into numerical and categorical variables. Categorical variables must be preprocessed by converting them into numerical variables. In this study, we use one-hot encoding [32] to represent categorical variables. This conversion transforms both discrete and continuous parameter types into numerical types. The one-hot encoding uses an *N*-bit state register to encode *N* categorical values, with each value having a corresponding register bit. The default value can be used for discrete or categorical parameters with only two possible values. The 22 parameter values in the form of K0, K1, K2 are combined to generate a training sample dataset St, consisting of 322 samples. However, it is desired to reduce the size of the training sample dataset. A representative final training sample dataset Ft is selected for the subsequent analysis step.

This study employs the PCA [16] algorithm as the preferred method for dimensionality reduction. This choice is based on its projection-based nature, its suitability for reducing the dimensionality of the training sample dataset, and its faster computational performance compared to alternative techniques. Accordingly, PCA is applied to reduce the dimensionality of the initial training sample dataset St. The specific procedure is outlined in Algorithm 2. The resulting dataset after dimensionality reduction is denoted as *Y*, which consists of 100 samples from the final training sample set Ft.
**Algorithm 2:** Dimensionality reduction method based on PCA for the initial training sample set.**Input**: Original samples $X=\{X_{1},X_{2},X_{3},\cdots ,X_{22}\}$, where each row represents    values of each parameter in the training samples and each column    represents the data of the *i*-th sample **Output**: The final sample dataset *Y***₁** Get the training sample dataset matrix *X*;**₂** Decenter *X* so that each parameter value is subtracted from the average of the three sample values of that parameter;**₃** Calculate the covariance: $XX^{T}/21$;**₄** Solving the eigenvectors and eigenvalues of covariance $XX^{T}/21$ by eigenvalue decomposition;**₅** Select the 100 eigenvectors with the largest eigenvalues and form the eigenvector matrix *Q* with the 100 eigenvectors corresponding to them as column vectors;**₆** Calculate Y=XQ, then the reduced-dimensional sample dataset is *Y*, which contains a total of 100 final training sample sets;**₇** Return Y.

### 4.2. Lasso-Regression-Based Performance Modeling

We select the parameter set according to the user manual for distributed message queues. However, given the unique requirements of the AIoT edge computing environment, certain parameters may have a more subtle impact on the performance of the distributed message queue compared to others. Consequently, it could be advantageous to further reduce the parameters based on the selected final training sample set Ft.

To construct a predictive model that accurately describes the throughput performance of our distributed message system, we employ Lasso regression [17] to determine parameter weights. This model is trained using the final sample training set, $F_{t}$, derived from the PCA dimensionality reduction applied in the previous stage. This $F_{t}$ comprises 100 final sampled datasets, ensuring the reliability of the results.

The original performance model, defined by Equation (1), comprises 22 parameters as features. Lasso regression is a linear regression method that incorporates an L1 penalty term into the loss function, promoting sparsity in the model. This penalty term steers the coefficients of less influential features towards zero, effectively excluding them from the model. The performance model is constructed following the process outlined in Algorithm 3.

(1) $$f_{a}\left(x\right)=\alpha_{0}+\alpha_{1}x_{1}+\alpha_{2}x_{2}+\cdots +\alpha_{22}x_{22}$$

**Algorithm 3: **Performance modeling and key parameters screening by Lasso regression.
**Input**: Preprocessed samples $Y=\{Y_{1},Y_{2},Y_{3},\cdots ,Y_{22}\}$
**Output**: Key parameters and their weightings
**₁** Get the preprocessed sample dataset *Y*;**₂** Configuring and running 100 sets of final parameter samples in a distributed message system;**₃** Test and obtain the throughput and status. Remove sample data that runs abnormally;**₄** Construction of a set of training data pairs consisting of parameter configurations and throughputs;**₅** Normalize the parameters to a normal distribution of N(0,1);**₆** Set the performance model $f_{a}\left(x\right)=\alpha_{0}+\alpha_{1}x_{1}+\alpha_{2}x_{2}+\cdots +\alpha_{22}x_{22}$ and the loss function $J\left(\alpha \right)=\frac{1}{200}\sum_{i-1}^{100}{\left(f_{a}\left(x^{\left(i\right)}\right)-y^{\left(i\right)}\right)}^{2}+\lambda \cdot \sum_{j-1}^{22}\left|\alpha_{j}\right|$;**₇** Update $\left[\alpha_{1},\alpha_{2},\cdots ,\alpha_{2}2\right]$ using gradient descent;**₈** Remove the parameters with small or zero absolute weights;**₉** Return parameters and their weights.


    Table 2 presents the configuration parameters of our proposed distributed message systems, accompanied by their corresponding weights that signify their influence on system performance. These parameters offer valuable insights into the crucial factors that exert a substantial impact on the performance of distributed message systems.

The Lasso regression analysis yielded a refined set of 14 parameters from the initial 22 adjustable parameters and their respective weights. With this reduced parameter set, we can now construct a performance model that accurately simulates the performance metrics of a distributed messaging system’s output in an edge environment, taking into account these 14 crucial parameter inputs. In the final step of DMSCO, we can employ this model to dynamically optimize the performance configuration, ensuring a seamless and efficient system. Equation (2) presents the final performance model to be optimized.

(2) $$\begin{array}{cc} f(x)= & \alpha_{0}+15.03x_{1}+70.35x_{2}+23.74x_{3}+25.16x_{4}+60.35x_{5}+124.32x_{6}-24.59x_{7}\\ \; & +70.42x_{8}+120.35x_{9}+54.36x_{10}+43.58x_{11}+73.66x_{12}-170.95x_{13}+34.32x_{14} \end{array}$$

where $x_{1}∼x_{14}$ are the key parameters and $\alpha_{0}$ is a constant with different sizes in different distributed message systems.

### 4.3. Optimization Method Based on Deep Deterministic Policy Gradient Algorithm

DDPG is a robust deep reinforcement learning algorithm [31] specifically designed to tackle continuous action spaces in complex, high-dimensional environments. It achieves this by blending deep Q-learning and actor–critic methodologies. The algorithm employs an actor network to generate actions and a critic network to estimate the Q-value function. To update the policy, it calculates the gradient of the Q-value function with respect to the policy parameters and utilizes it to update the actor network. DDPG incorporates techniques such as experience replay and target networks to enhance training stability and expedite learning.

**Figure 4:**
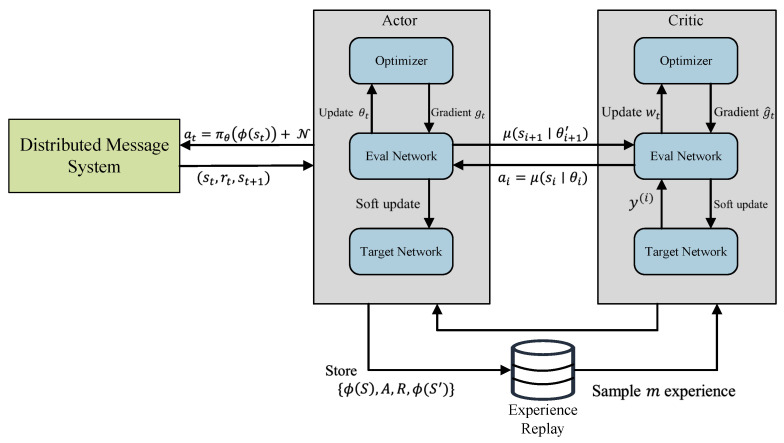
DDPG algorithm for distributed message system configuration optimization.

Two neural networks, namely the actor network and the critic network, are employed by the DDPG algorithm to handle continuous action spaces effectively [31]. To ensure the stability of the update process and mitigate the impact of continuous changes in the target, the DDPG algorithm utilizes separate current and target networks. This results in the involvement of four distinct neural networks: the current critic network, the current actor network, the critic target network, and the actor target network. The current actor network takes the current state as input and generates the action to be executed in the subsequent step. In contrast, the current critic network evaluates the present Q-value by taking action and state produced by the actor network as input and generates the corresponding Q-value as output. This separation of networks allows for effective learning and optimization in continuous action spaces. Figure 4 presents a schematic diagram illustrating the optimization using DDPG.

A comprehensive description of the algorithm is presented in Algorithm 4. To facilitate periodic updates of the two target networks, the DDPG algorithm employs a soft update strategy for the actor and critic target networks. This strategy involves gradually updating the target networks’ parameters using the parameters from their corresponding current networks. During the execution of the DDPG algorithm, the actor target network selects the optimal subsequent action, denoted as $A^{\prime }$, based on the next state $S^{\prime }$ sampled from the experience replay pool. At the same time, the critic target network computes the target Q-value.
**Algorithm 4: **DDPG-based distributed message system configuration optimization (DMSCO).
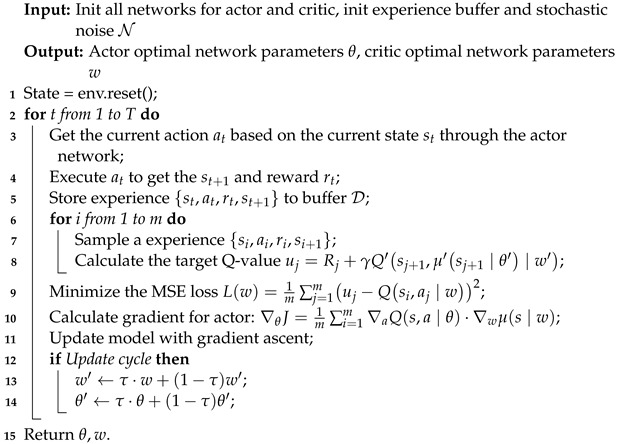


To begin, we initialize four deep neural networks: the evaluation critic network, evaluation actor network, target critic network, and target actor network. These networks utilize learnable parameters, denoted as *w*, θ, $w^{\prime }$, and $\theta^{\prime }$, respectively, to approximate the Q-value function and policy function.

At each time step, the current state $s_{t}$ and action $a_{t}$ are inputted into the current critic and actor networks, respectively, resulting in the estimation of the Q-value and the selected action. We then specify several hyperparameters, including the soft update coefficient τ, discount factor γ, experience replay buffer D, batch size *m* for batch gradient descent, target Q-network update frequency *C*, and the maximum number of iterations *T*. Additionally, a random noise function N is initialized to enhance learning coverage and introduce stochasticity during training. Finally, the first state in the state sequence, $s_{0}$, is designated as the initial state from which the learning algorithm proceeds. Following these initialization steps, we can train the DDPG algorithm to obtain an optimized policy for the given task.

To effectively employ the DDPG optimization framework, it is imperative to map the distributed message system optimization into a Markov decision process context.

- Environment: The environment refers to the distributed message system being optimized. We utilize the performance model built through the Lasso regression as the simulated edge environment, wherein the resultant increase or decrease in the system’s throughput serves as a performance-based reward.
- Agent: The configuration optimizer based on DDPG is regarded as the agent.
- Action: Action is depicted as a vector consisting of adjustable parameters.
- State: State can refer to the system running metrics.
- Reward: The reward is defined as the augmentation in throughput relative to both the initial configuration and the preceding one.

Furthermore, the primary parameters of the distributed message system are initially assigned default values, forming the initial state sequence *S*. A feature vector is computed to capture the parameter configuration of the current state. This feature vector represents the current state and is inputted into the actor network, which utilizes policy gradient techniques to update the network parameters.

Equation (3) [31] shows the determination of the appropriate action based on the network outputs. This equation is of significance as it reveals how our DMSCO algorithm generates an optimal configuration based on the current state and the learned policy. Implementing this configuration leads to a new system state that aids in updating the policy, thereby enabling the acquisition of an optimal approach.

(3) $$A=\pi_{\theta }\left(\phi \left(S\right)\right)+N$$

The policy function, denoted as $\pi_{\theta }$, is defined as a monadic function that indicates the most appropriate action *A* to be executed to maximize the reward corresponding to a specific input state ϕ(S). To update the parameter values of the distributed message system, the action *A* is executed, resulting in a new state. Subsequently, the reward *R* is computed based on the new state’s throughput compared to the previous state *S*. The new state is observed and its feature vector is obtained. The quadruple $S,\;A,\;R,\;S^{\prime }$ is then stored in the experience replay pool D. If the reward *R* equals 1, the current state is updated to $S^{\prime }$; otherwise, the current state remains unchanged. By employing this methodology, the distributed message system optimizes performance by assimilating prior experiences.

Our proposed DMSCO algorithm randomly samples 32 experience data from the buffer D, denoted as $\left\{\phi \left(S_{i}\right),A_{i},R_{i},\phi \left(S_{i}^{\prime }\right)\right\},i\in \left[1∼32\right]$; the target Q-value can be calculated as in Equation (4) [31].

(4) $$u_{j}=R_{j}+\gamma \cdot Q^{\prime }\left(\phi \left(S_{i}^{\prime }\right),\pi_{\theta }^{\prime }\left(S_{i}^{\prime }\right)|w^{\prime }\right)$$

Then, the algorithm minimizes the MSE loss in Equation (5) to update the critic evaluation network.

(5) $$L\left(w\right)=\frac{1}{32}\sum_{i=1}^{3}2{\left(u_{j}-Q^{\prime }\left(\phi \left(S_{i}\right),A_{i}|w\right)\right)}^{2}$$

Finally, DMSCO calculates the loss function in Equation (6). Update the actor network with gradient ascent [31] as in Equation (7):

(6) $$J\left(\theta \right)=-\frac{1}{32}\sum_{i=1}^{32}Q\left(\phi \left(S_{i}\right),A_{i}|\theta \right)$$

(7) $$\nabla_{\theta }J=\frac{1}{32}\nabla_{A}Q\left(S,A|\theta \right){{|}_{S=Q(\phi \left(S_{i}\right),A=\mu \left(Q\left(\phi \left(S_{i}\right)\right)\right))}\nabla_{w}\mu \left(S|w\right)|}_{s=Q(\phi \left(S_{i}\right))}$$

The iterative process continues until the maximum number of iterations, denoted as T=1000, is reached, resulting in the termination of the training process. The final output includes the parameter configuration associated with the optimal action $A^{*}$, which indicates the ideal configuration of the distributed messaging system specifically tailored to the current message transmission scenario.

### 4.4. Complexity Analysis

Our proposed DMSCO, as a configuration optimization algorithm for the system, consists of two stages: the parameter screening stage using the Lasso regression and the parameter value optimization stage using DDPG.

In the parameter screening stage, the computational complexity can be measured by the number of operations required to solve the optimization problem associated with Lasso regression. It is generally higher than ordinary least-squares regression but lower than other regularization methods such as Ridge regression. Factors influencing the computational complexity include the number of parameters *p* and the number of samples *n* in the dataset. We employ the least angle regression (LARS) algorithm to solve Lasso regression and it has a computational complexity of $O(np^{2})$.

In the parameter value optimization stage, we utilize DDPG (deep deterministic policy gradient) to refine the configuration of the distributed system, considering the integration of two distinct networks: the actor and critic networks. Each network includes a replica of the same model, referred to as the target network, enabling synchronization between the models. When considering the time complexity of the DDPG algorithm, it is crucial to account for the key components that contribute to its runtime. These components involve neural network calculations encompassing forward and backward passes, gradient descent optimization, and target network updates. The polynomial time required for forward propagation in the actor and critic networks is denoted as *A* and *C*, respectively, while the time for backward propagation is represented by $A_{\mathrm{grad}}$ and $C_{\mathrm{grad}}$, respectively. Furthermore, the polynomial time for model updates conducted by network optimizers, such as RMS and Adam, is denoted as Opt. The process of updating between evaluation and target strategies involves matrix replication, and the time for this operation is represented by Upd. The computational complexity of these steps can be expressed as follows:

- Forward propagation through the actor and critic networks occurs at each time step *t* within the range 1, 2, …, T. During these passes, we perform computations on the actor and critic networks. Assuming the complexity of the forward pass for the actor network is O(A), and for the critic network is O(C), the overall complexity for *T* time steps is O(T·(A+C)). It is important to note that the complexity of O(A) or O(C) depends on the specific size of the model, as these steps involve matrix multiplications.
- During each time step, a backward propagation is performed to calculate gradients for both the actor and critic networks. This step involves computing the gradients for the actor network with a complexity of $O(A_{\mathrm{grad}})$ and for the critic network with a complexity of $O(C_{\mathrm{grad}})$. Considering *T* time steps, the total complexity becomes $O(T\cdot \left(A_{\mathrm{grad}}+C_{\mathrm{grad}}\right))$.
- Gradient descent optimization involves performing the optimization process for each batch of size *B*. Considering the complexity of the optimization step as O(Opt), the overall complexity for *T* time steps can be estimated as O(T·Opt/B), as an optimization step is executed for every *B* time steps.
- The target networks are updated periodically every sync time steps, which is a hyper-parameter to control the frequency of synchronization. Assume the complexity of updating the target networks is O(Upd). This depends on the complexity of replication between two identical network matrices. The overall complexity for *T* time steps can be approximated as O(T·Upd/sync), as a target network update is performed for every sync time steps.

Taking all these components into account, the overall time complexity of the DDPG algorithm can be expressed as:

(8) $$O\left(T\cdot \left(A+C+A_{\mathrm{grad}}+C_{\mathrm{grad}}\right)\right)+O\left(T\cdot \mathrm{Opt}/B\right)+O\left(T\cdot \mathrm{Upd}/\mathrm{sync}\right)$$

The aforementioned analysis reflects the worst-case scenario of serial computation under typical circumstances. However, it is important to note that the actual complexity of the algorithm can be reduced by leveraging parallel acceleration techniques and optimization approaches. For instance, techniques such as pipeline computation for neural networks can be employed to enhance the overall efficiency of the algorithm. By harnessing parallelism and implementing optimization strategies, it is possible to improve computational performance and reduce the overall complexity of the algorithm in practical implementations.

## 5. Experiments

To evaluate the effectiveness of the proposed DMSCO algorithm, we create a simulated AIoT edge computing environment consisting of many edge devices. The specific configuration of the experiment environment is outlined in detail in Table 3.

The simulation environment comprises a cluster constructed using Docker containers, designed to simulate the AIoT edge computing environment. Messages within this environment are represented as matrix packets of varying sizes, with MD5 codes serving as checksum information. To simulate real-time messages, the producer container continuously generates data with normally distributed sizes and unconsumed identifiers, as long as the local buffer is not full. Subsequently, the MD5 checksum of each message is calculated. Upon receiving a message, brokers employ the proposed PSA to pass it into the corresponding partition. The message is then parsed based on the matrix address and size. This process on brokers generates a matrix packet containing the identity, sender ID, MD5 code, and matrix, which is subsequently sent to the consumer network port. If the transmission wait time exceeds the valid wait time, the transmission is assumed to have failed, and the failure is recorded. Finally, the consumer receives, parses, and checks the message, returning the checksum result for verification. To closely resemble a real AIoT network, the network setting also simulates the real AIoT communication environment. We utilized Docker network plugins (tc-htb and netem) to emulate various network conditions, such as latency, packet loss, and bandwidth limitations, in order to simulate 4G/WLAN devices in the edge environment. In particular, we set the latency at 100 ms and configured the bandwidth to 30 Mbps for uplink and 100 Mbps for downlink connections. We facilitated communication between containers by configuring each container with a unique IP address and using Docker’s bridge network configuration.

The experiments comprise three distinct procedures. Firstly, we assess the actual performance of the proposed method through multiple iterations of training optimization under various high-concurrency scenarios. Secondly, we compare the performance of our proposed DMSCO method with that of both the genetic algorithm (GA) and random searching using our distributed message queues. In these comparisons, we focus on AIoT scenarios characterized by high message frequency and small message sizes. Lastly, we examine the optimization efficacy of various methods in practical applications involving low message frequency and substantial message sizes. Through this comprehensive analysis, we can effectively evaluate the efficiency of our method in diverse situations.

### Analysis on Performance and Results

To evaluate the efficacy of our proposed DMSCO method, a series of experiments were conducted using a simulated environment consisting of 128 AIoT devices. In the first set of experiments, each device was programmed to transmit ten messages per second, with the size of each message following a normal distribution characterized by a mean (μ) of 128 KB and a standard deviation (σ) of 10. For the second set of experiments, the devices transmitted one message per second, with the size of each message conforming to a normal distribution characterized by a mean (μ) of 1280 KB and a standard deviation (σ) of 100. Through these carefully designed experiments, the efficiency of our method in optimizing the performance of large-scale AIoT message queuing systems under diverse conditions is thoroughly evaluated.

**Figure 5:**
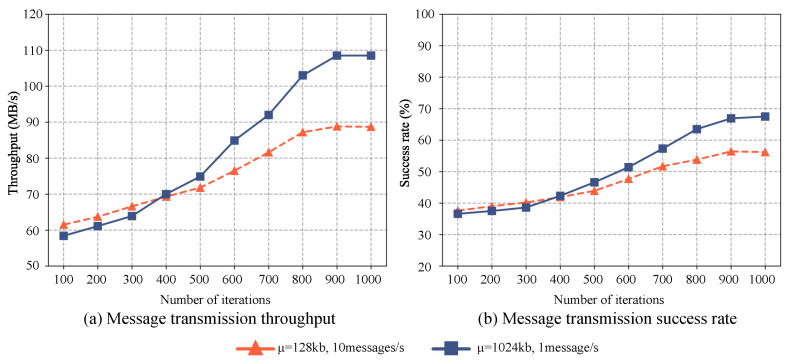
The performance of message transmission with different quantities and characteristics after optimized with DDPG. (**a**) illustrates the throughput variation and (**b**) illustrates the success rate variation.

As depicted in Figure 5, the optimized distributed message system demonstrates consistent improvement in the message transmission success rate across various high-concurrency scenarios. In the initial configuration, the IoT terminals transmit 10 messages per second, where the size of each message follows a normal distribution with a mean (μ) of 128 KB in the high-frequency small-message scenario. Comparatively, in the low-frequency large-message scenario, each terminal sends one message per second, and the size of each message adheres to a normal distribution with a mean (μ) of 1280 KB. The message transmission success rate is slightly higher in the low-frequency large-message scenario than in the high-frequency small-message scenario initially. However, as the iteration count approaches approximately 400, the success rates intersect, indicating their near equivalence. Beyond 400 iterations, a significant enhancement in the message transmission success rate is observed for the low-frequency large-message scenario, surpassing the performance of the high-frequency small-message scenario.

To validate the efficacy of the proposed DMSCO method, comparative experiments are conducted with the genetic algorithm and random-search-based method. In the case of the conventional genetic algorithm, the initial population is set to 100, the crossover probability to 0.5, and the mutation probability to 0.01. For the random-search-based method, random values are explored for each dimension of the key parameters.

**Figure 6:**
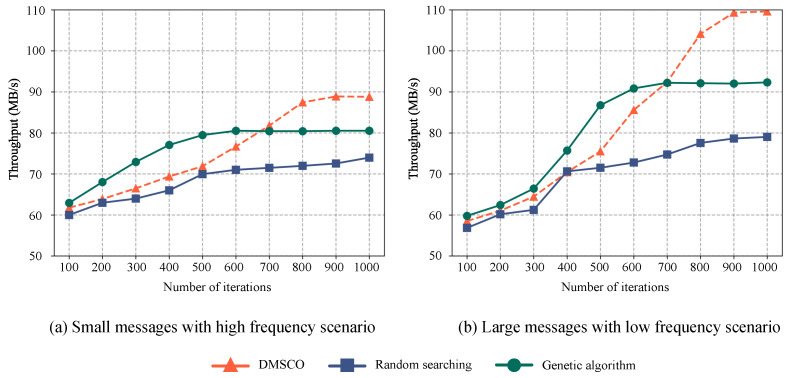
Optimization effects comparison of the proposed RL-based method, genetic algorithms, and random searching on distributed message systems in different concurrent scenarios. (**a**) Small message with high frequency. (**b**) Large message with low frequency.

Figure 6a presents a comparative analysis of message transmission throughput among edge terminals utilizing a distributed message queue. The experiments involve the transmission of 10 messages per second by each terminal, with message sizes following a normal distribution characterized by a mean (μ) of 128 KB and a standard deviation (σ) of 10. The objective is to evaluate the performance of different optimization methods in high message frequency and small message sizes scenarios. The results highlight the significant impact of the proposed DMSCO method, achieving an impressive optimized throughput of 88.79 MB/s, representing a remarkable improvement of 46.61% over the distributed message system without any configuration optimization. In comparison, random searching demonstrates a 22.17% improvement. The GA optimization method reaches its peak performance of approximately 80.52 MB/s after 600 iterations, exhibiting a notable improvement of 32.95%.

Figure 6b illustrates the variation in the throughput of message transmission in our proposed distributed message system. In this scenario, each terminal sends one message per second, with the size of each message following a normal distribution characterized by a mean (μ) of 1280 KB and a standard deviation (σ) of 100 KB. The results indicate that all three optimization methods positively impact the throughput of the distributed messaging queue in the scenario involving infrequent large messages. Over the course of 1000 iterations, both the DMSCO and the genetic-algorithm-based optimization method exhibit a higher overall improvement in throughput compared to the random-search-based method. Notably, after 900 iterations, the proposed optimization method surpasses the genetic algorithm optimization method, achieving the best result of 108.5 MB/s. To provide a comprehensive comparison, Table 4 presents the actual system metrics observed for various methods in the same simulation setup following the completion of the optimization process. The table demonstrates the optimization’s efficacy and efficiency by comparing their performance against the standard configuration of a vanilla Kafka, which serves as a baseline for comparison.

The experimental results highlight the superior performance of the proposed DMSCO method across various message scenarios. In high-frequency, small-message situations, our approach achieves a significant improvement of approximately 46.61% in both the message transmission throughput and overall performance compared to the default configuration after 1000 iterations. Conversely, in low-frequency, large-message situations, the method archives an increase of approximately 85% in the message transmission throughput and overall performance compared to the default configuration after 1000 iterations.

Furthermore, a comparative analysis of the experiments reveals that both the DMSCO method and the genetic algorithm exhibit substantial convergence and performance optimization effects when compared to random searching. Notably, the DMSCO method demonstrates improved throughput after approximately 700 iterations.

The genetic algorithm exhibits rapid convergence during the early stages of the optimization process. However, its rate of improvement gradually decreases thereafter. This phenomenon can be attributed to the premature emergence of a “super individual” during the mutation process, which causes the search process to become trapped in local optima. Various strategies [33] have been proposed to address the issue of local optima in genetic algorithms, including increasing the population density, utilizing multi-objective optimization techniques, and dynamically adjusting mutation probabilities. However, these approaches can be less efficient than DDPG, which is a sample-efficient method. DDPG can enhance performance and overcome local optima more effectively, making it a promising alternative in our proposed AIoT edge computing environment.

## 6. Conclusions

This study focuses on the challenges of performance and adaptability in distributed message queue systems in AIoT edge computing environments. Our investigations reveal that the system’s throughput and success rate can experience significant declines under high concurrency levels, underscoring the need for an effective optimization method. In order to address this issue, we introduce a partition selection algorithm (PSA) that effectively mitigates the adverse effects of message disorder in distributed messaging systems while enhancing the load-balancing capabilities of our proposed distributed messaging queue system. In addition, we propose DMSCO, a DDPG-based optimization method, to perform auto-configuration for the proposed distributed messaging systems in AIoT edge environments. DMSCO enables adaptive configuration adjustments to accommodate variations in message size, frequency, and device count. Experimental results demonstrate the superiority of DMSCO over traditional optimization methods, including genetic algorithms and random searching, across diverse messaging scenarios.

While DMSCO has demonstrated significant improvements in optimizing distributed message queue systems within AIoT environments, it is crucial to acknowledge and address any potential limitations to enhance its effectiveness further. One area of consideration is the inclusion of additional performance metrics, such as transmission latency and power consumption, which are pertinent factors in AIoT edge environments. By incorporating these metrics into the reward functions or performance models, more precise optimization directions can be pursued, leading to refined and comprehensive system enhancements. Furthermore, the methodology for constructing performance models in this paper does not incorporate an adaptive approach for accurately selecting from among hundreds of adjustable parameters, which currently necessitates manual selection. This aspect could be considered for future research.

## Figures and Tables

**Table 1:** Comparison of popular distributed messaging protocols for IoT networks.

Protocol	MQTT	CoAP	AMQP	HTTP
Communication Model	Publish/Subscribe	Request/Response	Publish/Subscribe	Request/Response
Lightweight	Yes	Yes	No	No
Bandwidth Efficiency	High	High	Medium	Low
Power Consumption	Low	Low	Medium	Medium
Real-Time Support	Limited	Limited	Yes	Limited
Security	Supplementary measures	Built-in options	Advanced options	Built-in options
Scalability	High	Medium	High	High

**Table 2:** Key parameters and their weights obtained by Lasso regression screening.

Parameter Name	Weight
bThreads	15.03
cType	70.35
nNThreads	23.74
nIThreads	25.16
mMBytes	60.35
qM·Requests	124.32
nRFetchers	−24.59
sRBBytes	70.42
sSBBytes	120.35
sRMBytes	54.36
acks	43.58
bMemory	73.66
bSize	−170.95
lMs	34.32

**Table 3:** Simulation environment setup for the DMSCO implementation.

Component	Specification/Version
Operating system	Ubuntu 20.04.1 LTS
CPU	48 CPUs—Intel Xeon Gold 6126 @ 2.60 GHz
Memory	187 GB
Hard drive	8.2 TB
LAN speed	10 GbE
Docker version	23.0.4
Framework	Springboot 2.7
Kafka image	wurstmeister/kafka:2.12-2.4.0
Kafka Java client	Producer and consumer
Maven libraries	spring-boot-starter-web: v2.1.4, spring-kafka:v2.1.7, lombok: 0.32-2018.2

**Table 4:** Final performance comparison between different optimization methods.

Methods	Scenario	Throughput	Success Rate
DMSCO	Small-size msg and high frequency	88.79 MB/s	57.78%
	Large-size msg and low frequency	108.50 MB/s	68.45%
Genetic algorithm	Small-size msg and high frequency	80.52 MB/s	49.80%
	Large-size msg and low frequency	92.32 MB/s	61.15%
Random searching	Small-size msg and high frequency	73.99 MB/s	45.38%
	Large-size msg and low frequency	79.03 MB/s	61.15%
No optimization	Small-size msg and high frequency	60.56 MB/s	39.54%
	Large-size msg and low frequency	58.51 MB/s	33.67%

## Data Availability

Not applicable.
